# The transcriptome of the mosquito *Aedes fluviatilis* (Diptera: Culicidae), and transcriptional changes associated with its native *Wolbachia* infection

**DOI:** 10.1186/s12864-016-3441-4

**Published:** 2017-01-03

**Authors:** E. P. Caragata, F. S. Pais, L. A. Baton, J. B. L. Silva, M. H. F. Sorgine, L. A. Moreira

**Affiliations:** 1 Grupo Mosquitos Vetores: Endossimbiontes e Interação Patógeno Vetor, Centro de Pesquisas René Rachou - Fiocruz, Belo Horizonte, Minas Gerais Brazil; 2 Grupo de Informática de Biossistemas e Genômica, Centro de Pesquisas René Rachou - Fiocruz, Belo Horizonte, Minas Gerais Brazil; 3 Instituto de Bioquímica Médica, Universidade Federal do Rio de Janeiro, Rio de Janeiro, Brazil

**Keywords:** *Aedes fluviatilis*, *Wolbachia*, Transcriptome, RNA-Seq, Metabolism, Mosquito, Vector control, Symbiont, Oxidative stress

## Abstract

**Background:**

*Wolbachia* is a bacterial endosymbiont that naturally infects a wide range of insect species, and causes drastic changes to host biology. Stable infections of *Wolbachia* in mosquitoes can inhibit infection with medically important pathogens such as dengue virus and malaria-causing *Plasmodium* parasites. However, some native *Wolbachia* strains can enhance infection with certain pathogens, as is the case for the mosquito *Aedes fluviatilis*, where infection with *Plasmodium gallinaceum* is enhanced by the native *w*Flu *Wolbachia* strain. To better understand the biological interactions between mosquitoes and native *Wolbachia* infections, and to investigate the process of pathogen enhancement, we used RNA-Seq to generate the transcriptome of *Ae. fluviatilis* with and without *Wolbachia* infection.

**Results:**

In total, we generated 22,280,160 Illumina paired-end reads from *Wolbachia*-infected and uninfected mosquitoes, and used these to make a *de novo* transcriptome assembly, resulting in 58,013 contigs with a median sequence length of 443 bp and an *N50* of 2454 bp. Contigs were annotated through local alignments using BlastX, and associated with both gene ontology and KEGG orthology terms. Through baySeq, we identified 159 contigs that were significantly upregulated due to *Wolbachia* infection, and 98 that were downregulated. Critically, we saw no changes to Toll or IMD immune gene transcription, but did see evidence that *w*Flu infection altered the expression of several bacterial recognition genes, and immune-related genes that could influence *Plasmodium* infection. *w*Flu infection also had a widespread effect on a number of host physiological processes including protein, carbohydrate and lipid metabolism, and oxidative stress. We then compared our data set with transcriptomic data for other *Wolbachia* infections in *Aedes aegypti*, and identified a core set of 15 gene groups associated with *Wolbachia* infection in mosquitoes.

**Conclusions:**

While the scale of transcriptional changes associated with *w*Flu infection might be small, the scope is rather large, which confirms that native *Wolbachia* infections maintain intricate molecular relationships with their mosquito hosts even after lengthy periods of co-evolution. We have also identified several potential means through which *w*Flu infection might influence *Plasmodium* infection in *Ae. fluviatilis*, and these genes should form the basis of future investigation into the enhancement of *Plasmodium* by *Wolbachia*.

**Electronic supplementary material:**

The online version of this article (doi:10.1186/s12864-016-3441-4) contains supplementary material, which is available to authorized users.

## Background

*Wolbachia pipientis* is a maternally inherited, bacterial endosymbiont that naturally infects an estimated 40% of terrestrial insect species [1]. *Wolbachia* induce a wide range of physiological manipulations in different insect hosts, with manipulation of reproductive biology promoting maternal transmission and thus bacterial propagation [2, 3]. It is through this ability to alter host biology that *Wolbachia* have gained interest as a form of biological control for the mosquito-transmitted pathogens that are responsible for diseases such as malaria, dengue fever, chikungunya and Zika fever, which represent a serious threat to human health across the globe [4, 5].

Many *Wolbachia* strains induce the reproductive manipulation cytoplasmic incompatibility (CI) in their hosts. This occurs when *Wolbachia*-infected male insects mate with uninfected females, which then produce unviable eggs. In contrast, *Wolbachia*-infected females successfully produce viable progeny after mating with either infected or uninfected males [3]. CI increases the proportion of *Wolbachia*-infected insects over subsequent generations, and serves to replace *Wolbachia*-uninfected individuals in population a population with those infected by the bacterium [6, 7]. CI-causing strains can be used to suppress mosquito populations that are uninfected by *Wolbachia* through the release of infected males, similar to the sterile insect technique, or to control *Wolbachia*-infected populations by releasing mosquitoes infected with a different strain, as this also crashes the population [8].

Several *Wolbachia* strains also produce anti-pathogenic effects in their hosts through the pathogen interference phenotype. The mechanics, scope and effectiveness of pathogen interference vary significantly between *Wolbachia* strains and insect hosts [9–12]. More effective pathogen interference severely inhibits pathogen development and transmission within the host. In *Aedes aegypti*, a prominent mosquito vector of human diseases, artificial *Wolbachia* infection interferes with the dengue, Zika, chikungunya, yellow fever and West Nile viruses, and other pathogens including the avian malaria *Plasmodium gallinaceum*, pathogenic bacteria, and the filarial nematode *Brugia pahangi* [13–19]. Recent work has also demonstrated that artificial *Wolbachia* infection in the malaria vector *Anopheles stephensi* can interfere with infection by the human malaria parasite *Plasmodium falciparum*, indicating that pathogen interference has broad applicability against human pathogens transmitted by mosquitoes [20].

CI and pathogen interference are the basis for the population replacement form of mosquito control utilised by the Eliminate Dengue Project (http://www.eliminatedengue.com/program). This strategy involves the release of *Wolbachia*-infected mosquitoes; CI allows the bacterium to spread and become stable within the target, wild population, while pathogen interference makes these mosquitoes less likely to transmit important viruses [17, 21]. *Wolbachia* has been successfully spread into a wild *Ae. aegypti* population [6], with the infection and strong pathogen interference against dengue virus persisting after several years of co-evolution [22, 23]

Neither *Ae. aegypti* nor *An. stephensi* are known to be naturally infected by *Wolbachia*. The infections of these mosquitoes described above were generated through transinfection, where *Wolbachia* is taken from a donor species and then injected into the eggs of the target species to create a stable germline infection transmitted to offspring [20, 24, 25]. In comparison to natural *Wolbachia* infections, such transinfections typically have a higher bacterial density, and infect a wider range of host tissues, which makes them far more likely to produce pathogen interference, and other extreme manipulations of host physiology [10, 20, 24]. Pathogen interference could potentially be lost from transinfected mosquitoes due to co-evolution between *Wolbachia* and host, or adaptation on the part of the pathogen [26]. Native *Wolbachia* infections typically produce minimal pathogen interference, and have little apparent utility to mosquito control strategies that require that trait. However, their low bacterial density, and presumed lower levels of virulence may be reflective the future biological state of transinfected mosquitoes after a long period of adaptation between host and symbiont.

Other native *Wolbachia* associations can enhance pathogen infection, as is the case for *w*Pip in *Culex pipiens* when challenged by *Plasmodium relictum* [27]. Enhancement is commonly associated with artificial transient somatic *Wolbachia* infections, and has not been seen with stable germline transinfections [2]. Its mechanism is unknown, but may involve changes to host immunity, metabolism or transcription [27–30]. Needless to say that both loss of pathogen interference, and the development of enhancement would be undesirable consequences if they were to occur in *Wolbachia* used for vector control. To that end, understanding how native *Wolbachia* strains influence host physiology at the molecular level will provide useful information about how these strains influence response to pathogens, and potentially highlight a mechanism for enhancement.

The mosquito *Aedes fluviatilis* inhabits non-urbanized regions throughout Latin America. It is not regarded as a vector for human pathogens in the field, however it is a good laboratory model for *P. gallinaceum* infection [31]. It is naturally infected by the *Wolbachia* strain *w*Flu, which grows only to low density, causes CI, and does not induce noticeable fitness costs [15, 32]. The effect of *w*Flu on dengue virus has not been investigated, however *w*Flu was shown to enhance *P. gallinaceum* oocyst numbers during some experimental infections [32], making it an interesting model to understand both native *Wolbachia* infections and pathogen enhancement. To determine whether there was a transcriptional basis for this enhancement and to further the understanding of native *Wolbachia* strains, and the extent to which they impact host biology, we used RNA-Seq to generate the transcriptome of *Ae. fluviatilis* mosquitoes both with and without their native *Wolbachia* infection.

## Results and Discussion

### RNA sequencing and de novo transcriptome assembly

We generated a total of 22,280,160 Illumina paired-end reads across 6 *Ae. fluviatilis* libraries - 3 with *Wolbachia* infection (*w*Flu), and 3 where the native infection had been cleared by treatment with tetracycline (Tet). Each library was sequenced from a pool of 16 whole adult female mosquitoes, collected 6 days after eclosion. After the trimming of adaptors and filtering for low quality reads we were left with 19,919,299 high quality paired-end reads (Q30% = 91), across all six libraries. As there was no published genome for *Ae. fluviatilis*, we attempted to map against the *Ae. aegypti* predicted transcriptome with Bowtie2, but less than 25% could be successfully mapped, which was unsuitable for further analysis. We then used the complete set of reads to make a *de novo* transcriptome assembly with Trinity (see methods). A total of 58,013 contigs were assembled encompassing 64 million base pairs (bp), with a median sequence length of 443 bp and an *N50* of 2454 bp (Table 1). Over 19,000 contigs were larger than 1Kb in size.

**Table 1:** Summary of sequencing results

Paired-end reads	Total base pairs	19,919,299
	Median length	171 x 2
	Q20 (%)	99
	Q30 (%)	91
*De novo* assembly	Total contigs	58,013
	Transdecoder ORFs	24,329
	Total bp in all contigs	64,257,972
	Median contig size (bp)	443
	Contigs larger than 1Kb	19,414
	N50	2454
Annotated contigs	NR	26,066
	KEGG	25,312
Mapping	Paired reads aligned concordantly 1 time (%)	66.78
	Overall alignment rate (%)	95.01

### Functional annotation

Annotation was performed through local alignments using BlastX (with an *e-*value of 1e-10) against the NCBI non-redundant database (NR), which returned annotation for 26,066 contigs. Of these, 14,209 contained unique hits (Table 1). As previous molecular characterization of *Ae. fluviatilis* has been limited to a few microsatellites [33], our analysis relied entirely on finding similarity to other genomes. Consequently, 31,947 contigs (55% of the entire data set) did not get a significant BlastX hit, which could be explained by misassembly or from lack of representation in the NR database. 920 of these were considered to be protein-coding sequences based on predictions made by Transdecoder, but no further characterization was possible for the remaining 31,027 contigs. For the purposes of our analysis we have assumed that gene function is identical to what was described in the blast hits, but this may not be the case, as genetic homology does not always imply functional homology. However, we are fairly confident given that majority of annotations were previously described in closely related mosquitoes.

**Fig. 1:**
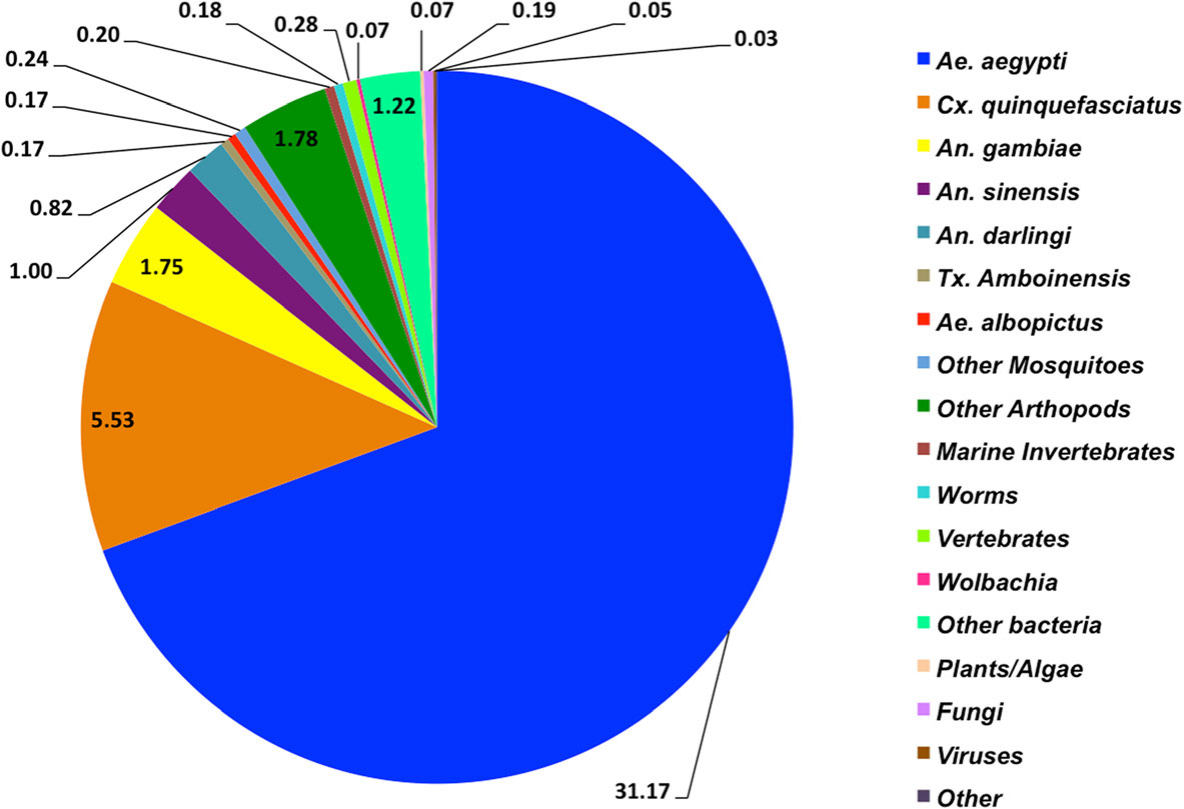
Breakdown of contig annotations by organism of origin. After *de novo* assembly, contigs were annotated by local alignment with the BlastX database. The chart depicts the percentage of contigs where the most significant BlastX hit matched to a particular species of clade. 90.93% of the hits were to a mosquito species. Contigs without a BlastX hit (31,951) are not shown

The assembled transcriptome of *Ae. fluviatilis* showed a high degree of similarity to *Ae. aegypti*, with 18,082 (69.38%) of the annotated contigs most closely matched to that species (Fig. 1). This was not surprising as *Ae. aegypti* has the most thoroughly annotated genome of the mosquitoes in the *Aedes* genus, meaning that this observed similarity may have more to do with the prevalence of *Ae. aegypti* sequences in public databases than the closeness of the relationship between the two species [34]. A further 5,616 (21.55%) of the annotated contigs were homologous to genes from other mosquito species, and overall 94.9% of the matches from BlastX were arthropod in origin. We conducted divergence analyses to better clarify the relationship between *Ae. fluviatilis* and other mosquito species (Additional file 1), and determined that *Ae. fluviatilis* diverged from these species approximately 98 million years ago (95% highest posterior density interval: 64.1 to 133.5 million years ago). A further 383 contigs matched to non-arthropod animals.

Across both the Tet and *w*Flu libraries we identified 751 contigs of bacterial origin (2.86%), and 112 of fungal origin (0.43%), which may potentially represent part of the *Ae. fluviatilis* intestinal microbiota, or could be the result of environmental contamination during sample collection. The bacterial sequences represented 250 distinct taxa, with the majority associated with a single contig. The diversity of sequence origins may indicate that the microbiota comprises many species, or that the majority of gut flora belonged to species that were not represented in the NR database. It is also possible that some of these contigs actually came from *Wolbachia*, but were different in composition from previously sequenced *Wolbachia* genes. Given the relatively low number of contigs of bacterial origin, the low copy numbers of these contigs and the low number of hits for each bacterial taxon in our data set, it was not possible to gain significant insight into the potential influence of *w*Flu on the composition or role of the microbiota.

Only 42 contigs of *Wolbachia* origin were identified in our data (Additional file 2). These contigs came from a variety of strains including *w*Ana, *w*Bm, *w*MelPop, and *w*Pip. 38 were expressed in the *w*Flu libraries, and 4 in the Tet libraries - potentially via integration into the host genome. *Wolbachia* genomes typically contain between 900–1400 coding sequences [35]. Of the 42 identified *Wolbachia* contigs, 22 were hypothetical proteins with unknown function. Interestingly, one contig was a heme biosynthesis protein, which suggests that *w*Flu promotes heme production, and provides further evidence to support the theory that *Wolbachia* alters host iron and redox homeostasis, even amongst native associations [36, 37]. We also identified a glycosidase hydrolase, which plays a role in sugar metabolism. The remainder of the *Wolbachia* contigs fell into two broad functional categories. The first included genes involved in DNA/RNA processing, DNA repair and RNA synthesis, and are likely part of normal *Wolbachia* replication and transcription processes, as similar genes have been identified in the *w*Mel genome [38]. A further group of contigs included *Wolbachia* membrane proteins, and ankyrin genes, which are used to attach the bacterial membrane to the host cytoskeleton. These genes likely represent part of the machinery used to mediate *Wolbachia*-host interactions [39], are typically present in large numbers in *Wolbachia* genomes [35, 38, 40–42], and may also be involved in CI [43].

The genomes of both *Wolbachia* strains and their insect hosts typically contain large quantities of mobile elements including transposons [38]. These can alter or disrupt the expression of genes, depending on their point of insertion into the host genome [34, 44]. We found 61 contigs related to transposable elements, none of which were matched to a *Wolbachia* genome. We also found a further 28 of viral origin, which could represent sequences from past or present members of the *Ae. fluviatilis* viral flora, which have not been well characterised.

**Fig. 2:**
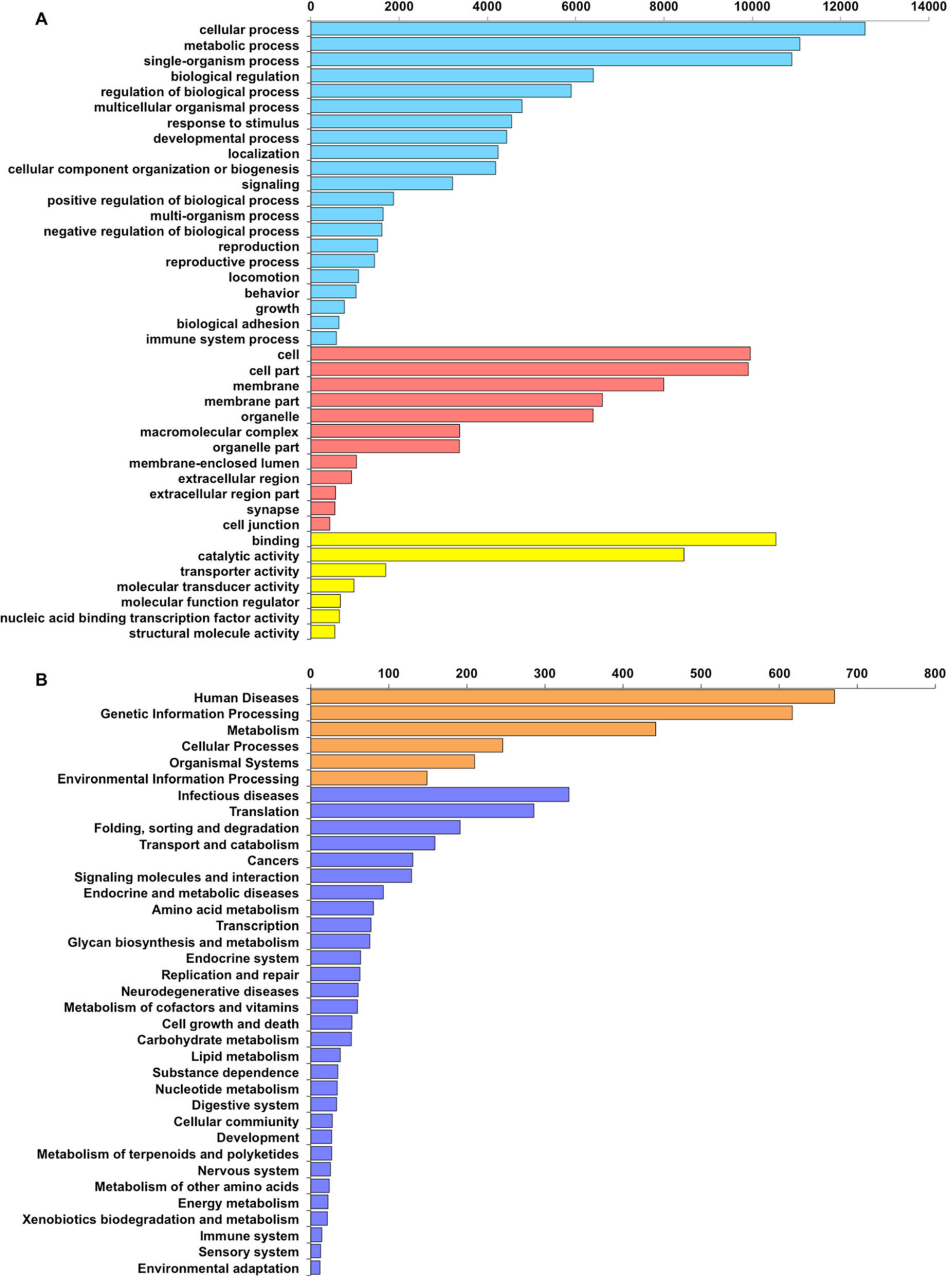
GO and KO terms associated with the *Aedes fluviatilis* transcriptome. **a** The 40 most enriched gene ontology terms in the biological process (blue), cell component (red) and molecular function (yellow) categories at level 2. **b** First level (orange) and second level (purple) KEGG orthology functional category terms associated with *Ae. fluviatilis*. GO and KO term lists were generated using combined data for both the *Wolbachia*-infected and -uninfected libraries

As part of the contig annotation process, gene ontology (GO) and KEGG orthology (KO) terms for each contig were identified using Blast2GO and the KEGG database, respectively (Fig. 2). We identified genes associated with a wide range of biological processes, functions and structures. From the whole set of contigs, 23,035 were assigned to at least one GO term. The majority of contigs in the biological process category were associated with cellular or metabolic process. Cell, and cell part were the cellular component terms with the greatest frequency, while binding and catalytic activity were the most common terms associated with the molecular function category. The most common KO terms were related to diseases, and molecular information processing.

### Differentially expressed contigs

Each RNA-Seq library was independently mapped to the assembled transcriptome using Bowtie. An average of 95% of the reads from each library were successfully mapped, and these data were used to generate counts for each contig. A list of differentially expressed contigs was generated using the baySeq package from Bioconductor, with 66% of these contigs (data not shown) also determined to be differentially expressed via analysis with the DESeq2 package, again from Bioconductor. Given this high level of concordance between the lists, we chose to proceed with further analysis of the baySeq list, as that method of analysis is known to be more sensitive [45]. Through baySeq we calculated the FPKM (Fragments Per Kilobase of transcript per Million mapped reads) for each contig, which considers the size of the contig in base pairs, and the overall data set size, as a measure of expression.

A total of 257 differentially expressed contigs were identified using baySeq, 159 were associated with the *w*Flu libraries, and 98 with the Tet libraries. Of these, 50 (19.4%) from the *w*Flu libraries (upregulated by *w*Flu) and 32 (32.7%) from the Tet libraries (downregulated by *w*Flu) had no matches after the BlastX search. This left 109 and 66 possible orthologs to known genes with increased and decreased expression due to *Wolbachia* infection, respectively (Additional file 3). Excluding multiple matches to genes with the same accession number, there were 95 unique contigs that were upregulated by *Wolbachia*, and 59 unique contigs that were downregulated, on the whole mosquito level. It is possible that analysis of whole mosquitoes precluded detection of genes that experienced a pattern of tissue-specific differential expression.

**Fig. 3:**
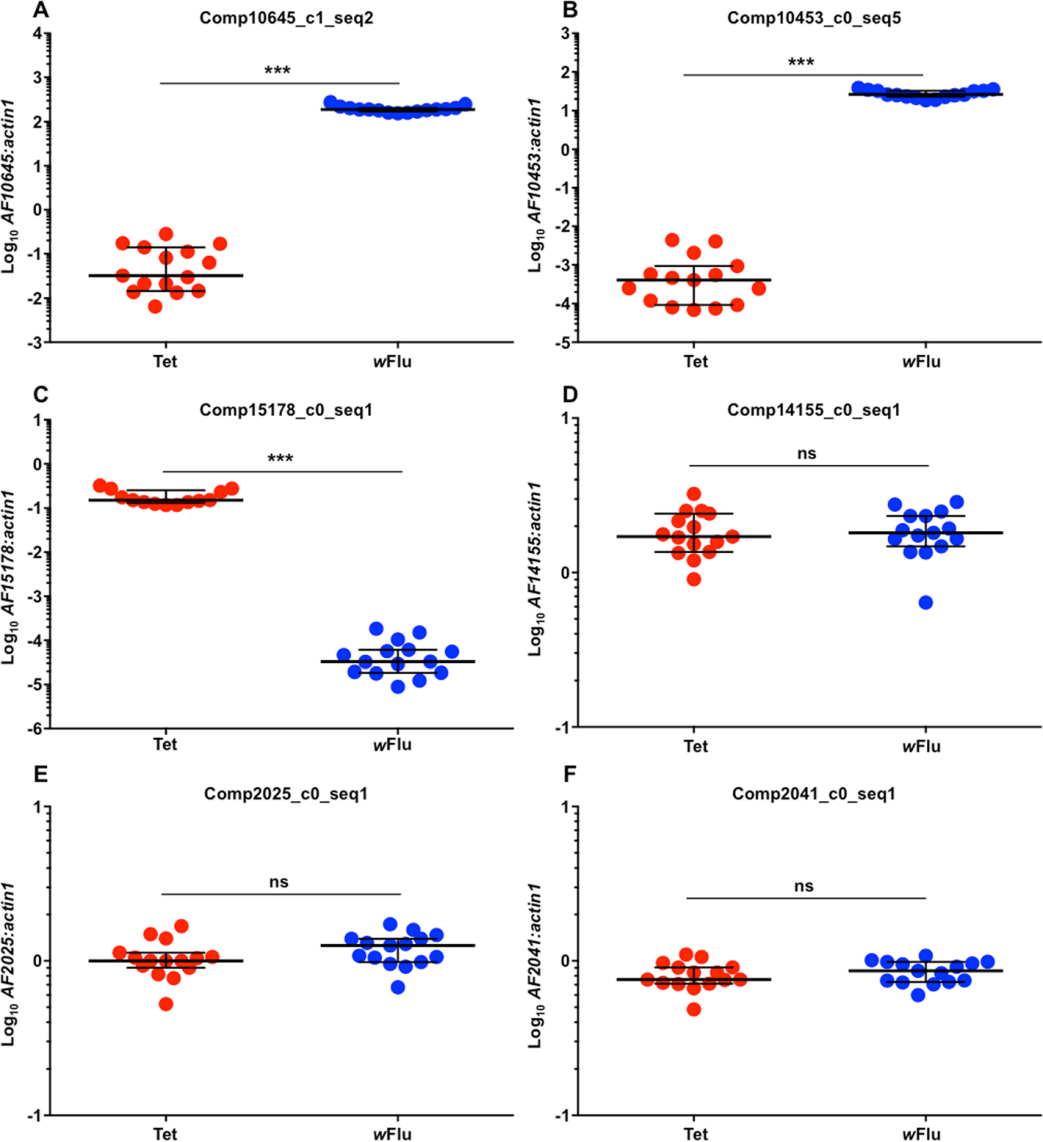
Confirmation of contig differential expression using RT-qPCR. 4 differentially expressed contigs (**a-d**), and 2 non-differentially expressed contigs (**e-f**) were selected at random, and expression levels were quantified via RT-qPCR. 5/6 contigs performed as expected, while one that was expected to show higher expression in *Wolbachia*-infected mosquitoes did not

To confirm the accuracy of the sequencing data, the expression of six contigs was examined using RT-qPCR (Fig. 3). Two contigs, comp10645_c1_seq2 and comp10453_c0_seq5 were predicted to be upregulated by *w*Flu, and further analysis confirmed that expression was significantly higher in *w*Flu mosquitoes (Mann Whitney U tests: 10645 - *P* < 0.0001; 10453 - *P* < 0.0001). A further 2 contigs were predicted to be downregulated by *w*Flu, and the first of these, comp15178_c0_seq1, demonstrated that pattern (Mann Whitney U test: *P* < 0.0001). However for the second, comp14155_c0_seq1, expression levels were not significantly different due to the presence of *Wolbachia* (Unpaired t test: *t* = 0.1196 *P* = 0.9057). All four of those contigs were predicted to display differential expression in both the bayseq and DESeq2 analyses. Only low levels of comp14155_c0_seq1 were detected during sequencing, which could explain the lack of differential expression observed during RT-qPCR. The final 2 contigs that were examined were predicted to have equivalent expression levels between treatments, and both fit that pattern (Unpaired t tests: comp2025_c0_seq1 - *t* = 1.500, *P* = 0.1449; comp2041_c0_seq1 - *t* = 0.7994, *P* = 0.4308). Further validation of the differential expression of these genes using different techniques will likely prove valuable for future research.

GO information for the annotated contigs was retrieved from Blast2GO, or repositories of transcriptional data such as FlyBase and VectorBase for some genes where Blast2GO produced no information (see methods). GO information could not be found for 28 upregulated contigs, and 17 downregulated contigs. The remaining upregulated contigs were associated with 286 GO terms, of which 93 terms had multiple hits. While downregulated contigs were associated with 190 GO terms, and 62 GO had multiple hits (Additional file 4). These GO terms and GenBank annotations were used to group the differentially expressed contigs based on their putative functions, and this information was used to develop profiles of the transcriptional changes that occurred both with (Table 2) and without (Table 3) *w*Flu infection. Some contigs had more than one annotated function, and are listed in multiple categories.

**Table 2:** Significantly upregulated contigs

Component number	Name	RPKM - *w*Flu	RPKM - Tet	Function
1. Membranes & membrane transport				
comp7819_c0_seq1	Niemann-pick type c2	268.16	88.12	Cholesterol transport
comp10576_c0_seq2	UNC93a protein	74.60	43.64	Transmembrane transport
comp13082_c0_seq1	Membrane transport protein, putative	52.03	16.70	Transmembrane transport
comp5926_c0_seq2	Monocarboxylate transporter	28.09	14.45	Transmembrane transport
comp9373_c0_seq2	CD36 antigen	12.48	5.80	Cell adhesion/lipid binding
comp6262_c1_seq3	Gustatory receptor 64a	9.64	2.24	Chemosensory receptor
comp9362_c0_seq1	Ionotropic receptor 100a	9.14	2.02	Chemosensory receptor
* comp14845_c0_seq1*	*OMPA-like protein*	*5.99*	*0.00*	*Wolbachia membrane*
* comp13777_c0_seq18*	*Permease*	*5.26*	*0.00*	*Transmembrane transport*
comp6505_c0_seq1	Glucose transport protein	2.15	0.22	Glucose transport
comp6457_c0_seq3	Sulfakinin receptor	1.19	0.25	Chemosensory receptor
2. Redox response				
comp13612_c0_seq1	Chorion peroxidase	287.11	168.42	Oxidative stress
comp8621_c0_seq1	Anterior fat body protein	135.03	79.59	Redox process
comp11151_c0_seq1	Cytochrome p450	81.07	29.11	Oxidase
comp8229_c0_seq2	Cytochrome p450	50.12	26.71	Oxidase
comp10103_c0_seq2	Neuferricin homolog, cytochrome b5 domain-containing protein 2	25.33	12.90	Heme binding
comp4282_c0_seq1	Cytochrome p450	9.14	3.10	Oxidase
comp6824_c0_seq1	Short-chain dehydrogenase	6.79	1.43	Oxidoreductase
3. Metabolism				
comp10525_c0_seq3	Brain chitinase and chia	196.30	85.85	Amino acid/carbohydrate metabolism
comp7163_c0_seq1	Chymotrypsin 1	179.35	69.13	Proteolysis
comp11118_c0_seq1	Gram negative bacteria binding protein 2	149.39	79.07	Carbohydrate metabolism
comp8621_c0_seq1	Anterior fat body protein	135.03	79.59	Lipoprotein
comp9990_c0_seq1	Phosphatidylcholine-sterol acyltransferase	87.08	32.92	Sterol/phospholipid metabolism
comp2787_c0_seq1	Serine protease	56.13	24.71	Proteolysis
comp4890_c0_seq1	Trypsin θ	47.87	12.96	Proteolysis
comp12424_c0_seq4	Pancreatic triacylglycerol lipase	45.47	19.55	Fat metabolism
* comp10453_c0_seq5*	*Orf16-lacz fusion protein*	*42.79*	*0.88*	*Carbohydrate/glycoprotein metabolism*
comp12444_c0_seq3	Glucosyl/glucuronosyl transferases	37.48	12.40	Carbohydrate/fatty acid metabolism
comp10786_c0_seq2	Serine protease	36.87	17.84	Proteolysis
comp13425_c0_seq4	Chitin metabolism protein	33.94	10.39	Peritrophic membrane metabolism
comp11198_c1_seq2	Serine 3-dehydrogenase	28.11	13.52	Amino acid metabolism
comp8292_c0_seq2	Galactose-specific C-type lectin	26.69	2.38	Carbohydrate metabolism
comp9458_c0_seq1	α chain crystal structure of β-glucosidase	18.76	5.39	Carbohydrate metabolism
comp12611_c0_seq1	β-hexosaminidase B	17.00	5.71	Carbohydrate/aminosugar metabolism
comp9289_c0_seq1	Serine threonine-protein kinase RIO1	16.31	3.07	Proteolysis
*comp13777_c0_seq6*	*PG1 homology to homo sapiens*	*14.47*	*0.55*	*Organic compound metabolism*
comp12709_c0_seq1	γ glutamyl transpeptidases	8.10	3.80	Amino acid metabolism
comp5863_c0_seq3	Acyltransferase	5.78	0.76	Protein modification
comp12058_c1_seq1	O-linked n-acetyl glucosamine transferase	5.75	0.27	Aminosugar metabolism
comp6912_c0_seq1	Glucosyl/glucuronosyl transferases	2.70	0.57	Carbohydrate/fatty acid metabolism
4. Signalling				
comp9906_c0_seq2	CDC42 protein	118.57	58.48	GTPase
comp8210_c0_seq1	Odorant-binding protein a10	49.07	17.86	Chemosensory protein serine/threonine kinase
comp8312_c0_seq6	GTP-binding protein di-ras2-like	24.21	13.00	GTPase
comp10042_c0_seq3	Stretchin- isoform v	5.39	0.27	Protein kinase
comp309_c0_seq1	ρ guanyl-nucleotide exchange factor	3.48	0.49	GTPase
5. Cell Process				
comp7767_c0_seq1	Protein frg1 homolog	69.85	39.25	rRNA processing/Gene silencing/activation
comp13326_c1_seq7	Histone h2b	49.34	27.23	Gene silencing/activation
comp10542_c0_seq1	Integrator complex subunit 12	27.74	10.71	snRNA processing
comp6248_c0_seq1	Chromobox protein homolog 1	20.33	7.35	Gene silencing/activation
*comp13777_c0_seq25*	*Ribosomal protein s10*	*13.45*	*3.44*	*Protein synthesis*
comp7336_c0_seq1	Hect e3 ubiquitin ligase	11.86	5.97	DNA repair
comp5798_c0_seq1	Zinc finger protein 425	10.96	2.44	Transcription factor
comp7025_c0_seq1	Muts protein homolog 5-like	10.24	5.38	DNA repair
comp8754_c0_seq1	Mitochondrial 28 s ribosomal protein s29	8.11	0.00	Translation
comp15539_c0_seq1	TcasGA2_TC002223 Zinc finger domain containing protein	4.07	0.00	Transcription factor
comp11749_c0_seq1	Muts protein homolog 4	3.58	1.18	DNA repair
comp2214_c0_seq1	RNase H and integrase-like protein	3.60	0.74	DNA replication & repair
comp13817_c0_seq21	RNA/mRNA processing protein	2.14	0.14	RNA/mRNA processing
6. Physiology				
comp13612_c0_seq1	Chorion peroxidase	287.11	168.42	Ovarian follicle maturation
comp5400_c0_seq1	Synaptic vesicle protein	37.67	20.32	Neurotransmitter trafficker
comp13425_c0_seq4	Chitin metabolism protein	33.94	10.39	Morphogenesis
comp11035_c0_seq1	Voltage-dependent para-like sodium channel	27.45	7.89	Nerve impulse
comp7184_c0_seq1	Fasciculation and elongation protein ζ-2	22.96	6.83	Signal transduction/neural development
comp14297_c0_seq1	Major allergen bla g	22.81	5.58	Neurotransmitter binding
comp11161_c0_seq1	Neuroblast formation protein	15.44	3.44	Cell division/neuroblast formation
comp11781_c0_seq19	BMP-induced, dendrite morphogenesis factor	2.75	1.08	Neural development
comp17984_c0_seq1	Voltage-dependent pq type calcium channel	2.36	0.00	Neurotransmitter release
7. Immunity				
comp11118_c0_seq1	Gram negative bacteria binding protein 2	149.39	79.07	Pathogen binding
*comp10645_c1_seq8*	*Cell wall-associated hydrolase*	*42.78*	*5.99*	*Bacterial cell wall degradation*
comp8292_c0_seq2	Galactose-specific C-type lectin	26.69	2.38	Pathogen binding
* comp14485_c0_seq1*	*Cell wall-associated hydrolase*	*12.27*	*1.61*	*Bacterial cell wall degradation*
8. Salivary Proteins				
comp4837_c0_seq1	Salivary basic peptide-1	259.44	69.40	Salivary protein
comp5901_c0_seq1	Anti-platelet protein	66.88	39.76	Salivary protein
9. Mobile Elements				
comp6660_c0_seq1	af378002_1 transposase	20.28	8.02	DNA integration
comp13085_c0_seq2	Reverse transcriptase	10.99	5.13	Reverse transcriptase
comp12175_c0_seq4	Retrovirus transposon polyprotein	7.68	3.39	Polyprotein
comp13891_c0_seq14	af378002_1 transposase	7.41	0.00	DNA integration
comp14791_c0_seq1	Uncharacterized protein K02A2	6.41	0.15	DNA integration
comp14916_c0_seq1	Retrovirus transposon polyprotein	4.76	0.00	Polyprotein
*comp13573_c0_seq26*	*Endonuclease-reverse transcriptase*	*1.20*	*0.10*	*Reverse transcriptase*

Contigs in italics are of bacterial origin. FPKM - Fragments Per Kilobase of transcript per Million mapped reads for the wFlu and Tet libraries

**Table 3:** Significantly downregulated contigs

Component number	Name	RPKM - *w*Flu	RPKM - Tet	Function
1. Membranes & membrane transport				
comp7683_c0_seq2	Membrane glycoprotein lig-1	7.27	22.40	Membrane receptor
comp3093_c0_seq1	EGF-like module-containing mucin-like hormone receptor-like 1	2.09	11.49	Membrane receptor
*comp1729_c0_seq1*	*ABC transporter ATP-binding protein*	*0.33*	*4.41*	*Transmembrane transport*
2. Redox response				
comp5605_c0_seq1	Anterior fat body protein	16.34	36.66	Redox process
comp8781_c0_seq2	Dimethylaniline monooxygenase	0.42	2.93	Redox process
3. Metabolism				
comp5123_c0_seq1	Serine protease 14	85.21	201.81	Proteolysis
comp8292_c1_seq1	Galactose-specific C-type	66.95	145.97	Carbohydrate metabolism
comp10957_c0_seq1	Serine protease	47.69	111.75	Proteolysis
comp8332_c0_seq1	Diacylglyceride synthesis protein	20.73	48.52	Fat synthesis
comp5605_c0_seq1	Anterior fat body protein	16.34	36.66	Lipoprotein
comp7785_c0_seq8	Glucosamine 6-phosphate N-acetyltransferase, putative	10.17	23.93	Amino acid/aminosugar metabolism
comp14516_c0_seq1	Zinc metalloprotease	0.71	12.68	Proteolysis
comp9411_c0_seq5	Carboxylesterase	1.78	7.61	Hydrolase
comp2544_c0_seq1	Vitellogenic carboxypeptidase	0.80	6.73	Proteolysis
comp16467_c0_seq1	Zinc metallopeptidase	0.00	4.09	Proteolysis
comp17222_c0_seq1	Acyltransferase	0.25	3.39	Protein modification
4. Signalling				
comp9906_c0_seq1	CDC42 protein	11.93	69.66	GTPase
comp11677_c0_seq3	Dual specificity tyrosine-phosphorylation-regulated kinase	13.96	31.43	TPR Kinase
comp7548_c0_seq2	Calmodulin-like protein 4-like	13.13	30.04	Signal transduction
comp5282_c0_seq1	Signal peptidase complex subunit 1	5.76	26.11	Signal protein cleavage
comp11677_c0_seq2	Dual specificity tyrosine-phosphorylation-regulated kinase	13.17	25.49	TPR Kinase
comp10289_c0_seq6	Guanine nucleotide-binding protein subunit	5.47	16.12	Signal protein
comp9283_c0_seq2	GTP-binding protein alpha gna	1.08	4.54	GTPase
5. Cell Process				
comp14155_c0_seq1	T-complex polypeptide 20	0.94	25.10	Protein folding
comp8487_c0_seq1	Histone h1	6.28	18.53	DNA binding
comp4291_c0_seq2	RNase h and integrase-like protein	3.25	15.80	DNA repair
comp6475_c0_seq2	Insulin receptor tyrosine kinase substrate	0.21	3.09	Cytoskeleton organization
6. Physiology				
comp13996_c0_seq1	Rhodopsin receptor 1	3383.34	7140.82	Visual receptor
comp9690_c0_seq8	Photoreceptor protein	1087.17	2438.80	Phototransduction
comp8001_c0_seq1	Ultraviolet-sensitive opsin	78.20	207.73	Visual receptor
comp12700_c0_seq1	Calmodulin-binding protein trpl	41.91	101.60	Phototransduction
comp9621_c0_seq1	Blue-sensitive visual pigment	16.68	56.19	Phototransduction
comp3173_c0_seq1	Rhodopsin	19.70	51.63	Visual receptor
comp2653_c0_seq1	Mucin-like peritrophin	11.81	39.14	Petritrophic matrix component
comp8706_c0_seq1	Cuticular protein isoform a	1.62	10.60	Cuticle component
comp4000_c0_seq1	Photoreceptor protein	0.00	8.21	Phototransduction
comp5558_c0_seq1	Hypothetical protein ZHAS	0.43	3.63	Cell growth
7. Immunity				
comp8292_c1_seq1	Galactose-specific C-type lectin	66.95	145.97	Pathogen binding
comp3093_c0_seq1	EGF-like module-containing mucin-like hormone receptor-like 1	2.09	11.49	Immune signalling
8. Salivary proteins				
comp6651_c0_seq1	Short form d7 salivary protein sd7-1	39.94	79.53	Salivary protein
comp6296_c0_seq4	Short form d7 salivary protein sd7-1	4.35	18.17	Salivary protein
9. Mobile elements				
comp14966_c0_seq1	af377999_1 transposase	1.08	6.80	DNA integration
* comp334_c0_seq2*	*line-1 retrotransposon-like*	*0.00*	*6.43*	*DNA integration*
comp4558_c0_seq2	bel12_ag transposon polyprotein	1.60	5.48	Polyprotein
comp13589_c0_seq12	Uncharacterized protein K02A2.6-like	1.25	5.23	DNA integration
comp4787_c0_seq1	bel12_ag transposon polyprotein	1.16	4.89	Polyprotein

Contigs in italics are of bacterial origin. FPKM - Fragments Per Kilobase of transcript per Million mapped reads for the wFlu and Tet libraries

### Immune stimulation and suppression

Transinfection with *Wolbachia* in mosquito species has typically led to widespread increases in the expression of immune genes, including those involved in the Toll and IMD immune pathways, and a large number of antimicrobial peptides [10, 11, 14, 20]. This immune activation has been linked to interference of dengue viruses in mosquitoes [10, 14, 15], and immune suppression has been hypothesized as a potential cause of the enhancement of *Plasmodium* infection seen in some native and transient *Wolbachia* infections [28, 29]. We observed no changes in the transcription of genes in the Toll or IMD pathways, including genes such as *rel1*, or *rel2*, which might have been indicative of a change in pathway regulation. We also saw no changes in the expression of any of the antimicrobial peptides commonly affected by *Wolbachia*. The absence of systemic immune activation is common amongst native *Wolbachia* infections, and may be symptomatic of increased tolerance on the part of the host, and reduced pathogenicity on the part of the symbiont [46, 47].

We did observe differential expression of 6 contigs directly involved in mosquito immunity, 4 upregulated (2 cell wall hydrolases, a galactose specific c-type lectin, and a gram negative bacteria binding protein) and 2 downregulated (a mucin like protein, and a galactose specific c-type lectin). These genes are typically associated with bacterial binding and degradation, but many have also been linked to *Plasmodium* infection and could have contributed to the enhancement of *P. gallinaceum* infection in *Ae. fluviatilis* [32]. Gram negative bacteria binding proteins, for instance can have a broader role in immune stimulation, and are involved in the response to *Plasmodium* infection in *An. gambiae* [48]. Some galactose-specific c-type lectins are highly differentially expressed by *Wolbachia* [11], their expression is stimulated by *Plasmodium* infection, and they have been shown to protect *Plasmodium* against melanisation by the host immune system [49]. There are also examples of mucin like genes and cell wall hydrolases that are critical to *Plasmodium* development in mosquitoes [50, 51].

We also identified other differentially expressed genes have also been linked with insect immunity or *Plasmodium* infection. *Plasmodium* infection is dependent on glycolysis, and the metabolism of amino acids and lipids [52], all affected by *w*Flu infection (see below). There was evidence of altered sugar metabolism and transport, which could promote the development of *Plasmodium* [53]. Additionally, there was increased expression of a β-hexosaminidase, and these genes have a peptidoglycan hydrolase effect, can be bactericidal, and have a broader immune function that could facilitate interaction with *Plasmodium* [54]. Finally, we also saw upregulation and downregulation of serine proteases, which are involved in both digestion and immunity. Serine proteases have been linked to *Plasmodium* growth and development, invasion of host cells, and can protect the parasite against the host oxidative stress response [55, 56]. Any of these transcriptional changes could be linked to the enhancement of *P. gallinaceum*, and therefore merit further investigation.

### Redox process

One interesting characteristic of *Wolbachia* infection is the breakdown of redox homeostasis, which has been seen across multiple infected hosts [57, 58]. This effect often manifests through the induction of reactive oxygen species (ROS), and altered expression of genes involved in oxidative stress response, which occur with both native *Wolbachia* associations and transinfections [10, 12, 58], but it is unclear if they represent an immune response to *Wolbachia*, or normalization of the redox processes altered by infection [37]. Increased oxidative stress has been linked to pathogen interference against viruses and *Plasmodium* [10, 11, 20], and may be part of *Wolbachia*’s immune evasion strategy [37]. Similarly, higher oxidative stress levels are an important part of the anti-*Plasmodium* immune response [59].

In *Ae. fluviatilis* we saw evidence of an altered redox response due to *w*Flu infection, in the form of higher levels of a short-chain dehydrogenase, an oxidoreductase, which can induce ROS, and 3 cytochromes p450, which act as oxidases [60, 61]. *Wolbachia* have also been linked with iron metabolism and storage, and this can influence key physiological traits such as fecundity [62–64]. *w*Flu infection induced higher levels of neuferricin, a protein that binds iron-rich heme, which is interesting given that *Wolbachia* produce enzymes involved in heme synthesis [38], and may utilise it as an energy source [36]. Likewise, heme is essential to the development of *Plasmodium* in mosquitoes [65]. Given that *w*Flu appears to alter redox homeostasis, it would be interesting to see if *w*Flu infection induces higher ROS levels. It should be noted that tetracycline treatment impacts mitochondrial function, which can lead to changes in insect oxidative stress response [66]. While we did use tetracycline to clear the *w*Flu infection, our experiments were performed more than 2 years (approximately 30 generations) after antibiotic treatment.

### Metabolism

We observed that 33 genes associated with metabolism and digestion were differentially expressed as a result of *w*Flu infection. *Wolbachia* demonstrate many nutrition-based physiological changes in different insect hosts, including mutualism through nutritional provision [40, 67], better performance under nutritional stress [62], and competition for nutrients leading to effects on host fecundity and fertility, and on pathogen interference [68, 69]. These processes are linked to a variety of nutrients and micronutrients including amino acids, iron, and flavin adenine dinucleotide, which indicates that *Wolbachia* interact with a wide range of host metabolic pathways.

*Wolbachia* infections in *Ae. aegypti* show differential expression of large numbers of digestive enzymes, particularly serine proteases and trypsins, which may indicate cannibalisation of host resources to promote *Wolbachia* propagation. Infection with *w*Flu increased the expression of 3 serine proteases, and 2 trypsins, and decreased levels of two other serine proteases and 2 zinc metallopeptidases. While the number of affected genes were fewer than seen for the *Ae. aegypti* transinfections it is sufficient to suggest that a similar process operates in *Ae. fluviatilis*. Likewise, *w*Flu infection elevated levels of brain chitinase and chia, an enzyme involved in breakdown of the peritrophic matrix, which may be further evidence of an altered digestive process, and could compromise the integrity of the peritrophic matrix and potentially facilitate *Plasmodium* invasion.

We observed that *w*Flu infection had a broad effect on several aspects of host metabolism, including carbohydrate and lipid metabolism, both areas where *Wolbachia* is lacking key biosynthesis genes, and where *Wolbachia* transinfection alters transcription in *Ae. aegypti* [11, 38, 70]. Previous work indicates that *w*Flu infection leads to elevated levels of glycogen [71], a major carbohydrate reserve, in developing embryos. While it is unclear if this effect occurs in adults, we did see elevated levels of β-glucosidase, which encodes an enzyme involved in the breakdown of complex carbohydrates including glycogen. Similarly, β-hexosaminidase b levels were also higher, with this enzyme involved in the hydrolysis of amino-sugars. *Wolbachia* heavily utilise host lipids and sterols, and alter the cellular lipid profile [68, 72, 73], potentially to serve bacterial propagation [74], with similar processes underlying *Plasmodium* development [75]. Lipid metabolism genes affected by *w*Flu had a diverse range of functions and include glucosyl glucuronosyl transferases, enzymes required for glucuronidation, which has a role in the metabolism of fatty acids and other compounds, and also pancreatic triacylglycerol lipases, which are used to digest triacylglycerides and process fats. Interestingly, *w*Flu also increased levels of phosphatidylcholine-sterol acyltransferase, which is involved in sterol and phospholipid metabolism, and may be utilised by *Wolbachia* to fabricate its membranous vacuoles. *w*Flu infection also decreased levels of diacylglyceride (DAG) synthesis protein, suggesting that the infection likely interferes with DAG synthesis, and therefore lipid transport as DAGs are prominent transport lipids. Likewise, changes in the expression of these genes could potentially promote *P. gallinaceum* development.

Infection also induced expression of chorion peroxidase, an enzyme involved in ovarian follicle maturation, and decreased the levels of a vitellogenic carboxypeptidase, which is a yolk protein produced in the fat body [76]. The effect on these genes likely arises due to the strong presence of *w*Flu in the ovaries, and may contribute to further differences in egg nutritional content, as is seen with glycogen [71].

### Membranes

We observed that infection with *w*Flu also affected 11 transcripts involved in membrane structure, or transmembrane transport. This included an ompa-like protein that is part of the *Wolbachia* membrane, which was indicative of *Wolbachia* replication. We also saw the upregulation of membrane proteins involved in the transport of glucose and cholesterol through niemann-pick type c2 protein, and these could potentially be involved in altering *Plasmodium* infection dynamics, given the varied metabolic requirements of the parasite [77]. This is further evidence of a co-opting of metabolic machinery, as *Wolbachia* has been strongly linked to carbohydrate metabolism as an energy source [38], and cholesterol, as a critical component of the vacuoles that surround the bacteria [78].

Infection also caused the upregulation of 3 membrane-bound chemosensory receptors potentially linked to *Ae. fluviatilis* behaviour. These three include sulfakinin, a digestive regulator in insects [79], and 2 taste receptors, including an ionotropic receptor and a gustatory receptor linked to sugar feeding [80]. *Wolbachia* infection can influence host olfaction at the behavioural [81, 82] and molecular level [11], and in the case of *w*MelPop, also dramatically alters feeding behaviour [83]. These effects could possibly contribute to a minor change in olfactory response or potentially behaviour in response to *w*Flu infection. In contrast, only 3 membrane-related genes were differentially expressed in the Tet dataset, including an ATP-dependent transporter molecule of bacterial origin, and two membrane receptors with putative roles in immune signalling, and membrane-protein interactions.

### Physiology

We saw that *w*Flu upregulated several genes with a potential role in neurological function and development. These included calcium and sodium channels, a major allergen, a fasciculation and elongation protein, which is involved in nerve signal transduction, and neuroblast formation protein, and BMP-induced dendrite morphogenesis factor, which are both involved in neural development. While *w*Flu does not heavily infect many non-reproductive tissues, it is present in the head at low density [15], and so could affect neurological function. Both *w*Mel and *w*MelPop infect neural tissues [15, 17], and *w*MelPop has a pronounced effect, causing neurological degradation in *Drosophila melanogaster* [84]. *Wolbachia* also affect hormone levels and contribute to host behavioural changes [85–87]. The overall physiological effect of *w*Flu infection is unclear, however given that genes involved in neurological development, signalling and neurotransmitter trafficking and release were all upregulated by *w*Flu, there could be critical effects.

While *w*Flu does infect *Ae. fluviatilis* heads, it is not found in the omatidia cells of the eye, unlike *w*MelPop [15], yet *w*Flu infection decreased the expression of 4 photoreceptor proteins involved in phototransduction, and 3 visual receptors, indicating that *w*Flu may influence host visual perception. There have been no categorised effects of *Wolbachia* on host visual process, and no similar genes were affected by *w*Mel or *w*MelPop [11]. Potential physiological consequences of these changes could be a decreased sensitivity to light, which would be disadvantageous to the mosquito, or may indicate decreased activity during low level light conditions [88–90].

### Cellular processes

There was also evidence that *w*Flu infection altered common cellular processes by influencing the expression of genes involved in DNA repair and replication, and DNA packaging (Tables 2 and 3), with similar genes affected by other *Wolbachia* infection in mosquitoes - particularly *w*MelPop [11]. Other contigs were also linked to the processes of transcription and translation, and more specifically to mRNA processing, snRNA and rRNA processing, and protein folding, which is not unexpected given that *Wolbachia* produces and affects the production of small RNAs [91, 92].

We observed that *w*Flu altered the expression of chromobox protein homolog 1, a heterochromatin protein that could potentially be involved in epigenetic silencing of gene expression, or in chromosome integrity. Likewise, we observed altered levels of histone 2b, which is involved in DNA packaging as a key component of the nucleosome, and in gene silencing. *Wolbachia* infection in *Ae. aegypti* mosquitoes is known to affect cytosine methylation and gene silencing [93], and our results could indicate that there could potentially be a similar influence on silencing through histone modification. In that study, *Wolbachia* strongly altered the methylation profile of genes involved in membrane function including transport, and here we observed that a similar group of genes were affected during *w*Flu infection in *Ae. fluviatilis*.

Reproductive manipulation in *Wolbachia*-infected insects has also been associated with histones and chromatin. For instance, CI has been linked to delays in the deposition of histones in the male pronucleus [94], while male-killing has been linked to defective chromatin packaging, and altered chromosome behaviour [95]. Critically, the molecular effects of these processes are associated with adult males (CI), or in early stage embryos, and while our data were generated from adult females, these results may suggest that there are broader effects of *Wolbachia* infection on cellular processes related to DNA packaging, chromatin formation, and the regulation of gene expression in mosquitoes.

### Comparison to other Wolbachia infections in mosquitoes

As *w*Flu is a native *Wolbachia* infection, and has likely undergone a lengthy period of co-evolution with *Ae. fluviatilis*, we were quite surprised to discover that infection affected the expression of genes involved in a wide range of host biological processes. The scope of the transcriptional changes associated with infection (257 genes), appears to be similar to that of *w*Mel in *Ae. aegypti* (327 genes), although that study utilized a microarray rather than RNA-Seq, and the difference in sensitivity of the techniques may be a factor [11]. This similarity is interesting given that the latter association is both relatively novel, and is a transinfection that produces a broader range of physiological effects, and has greater bacterial density [15, 17, 32]. In contrast, transinfection of *Ae. aegypti* with the more virulent *w*MelPop strain has been demonstrated to alter the expression of 244 genes in one study [14], and 2723 in another [11], with the studies utilizing different methods of analysis. A larger set of differentially expressed genes would likely reflect the pathogenicity associated with *w*MelPop infection.

While these *w*Mel and *w*MelPop data come from transinfections in a different species, we sought to determine if there was a core set of genes or functional groups of genes that were differentially expressed in all three associations, as this might explain something fundamental about the nature of *Wolbachia* infections in mosquitoes. 75 of the upregulated contigs, and 54 of the downregulated contigs were homologous to previously described *Ae. aegypti* transcripts in VectorBase. However, only 42 of these were also differentially expressed during either *w*Mel or *w*MelPop infection. Interestingly, the majority of these were upregulated by *w*Mel and *w*MelPop, even if they were downregulated by *w*Flu (Additional file 5), with this difference likely due to the relative novelty of the former transinfections.

Taking a broader approach, we then compared types of genes affected by all three strains, for example looking at all serine proteases, rather than a specific serine protease. Forty-six of the same types of genes were affected by both *w*MelPop and *w*Flu, with 33 upregulated and 13 downregulated (Additional file 5). For *w*Mel and *w*Flu, 15 genes of the same type were affected, with 12 upregulated and 2 downregulated, all of which were also affected by *w*MelPop (Table 4). Genes that are upregulated in the presence of *Wolbachia* reflect 5 key areas, protein and fat metabolism, redox process, membrane transport, DNA/RNA processing, and bacterial recognition, all of which have been previously characterised in *Wolbachia* infections [10, 11, 38, 72, 96]. Genes that were downregulated include cuticle proteins and carboxypeptidases, which are involved in protein digestion. Cuticle proteins can be downregulated in response to tetracycline treatment in *Wolbachia*-infected *Brugia malayi* worms [97]. They can also be downregulated in response to viral infections, and potentially play a role in host resistance to infection [98]. These processes likely contribute to making the mosquito host environment more favourable for *Wolbachia* propagation, and thus represent areas of interest for further study.

**Table 4:** Core genes affected by *Wolbachia* infection in mosquitoes

Upregulated	Downregulated
Cytochrome p450	Carboxypeptidase
Galactose-specific C-type lectin	Cuticle protein
Gram negative binding protein	
Membrane transport protein	
Odorant binding protein	
Pancreatic triacylglycerol lipase	
Permease	
Serine protease	
Serine/Threonine protein kinase	
Short-chain dehydrogenase	
Trypsin	
Zinc finger protein	

## Conclusions

Here, we present the transcriptome of the mosquito *Ae. fluviatilis*, and consider the transcriptomic effects of its native *Wolbachia* strain, *w*Flu. Previous results suggest that *w*Flu infects host tissues at relatively low densities, causes incomplete CI and has no observable fitness cost [15, 32], in accordance with theories that suggest native *Wolbachia* strains have lost bacterial density and pathogenicity during long periods of co-evolution, and the development of tolerance on the part of their hosts [26]. Our data indicated that *w*Flu infection led to the differential expression of 257 genes, and while the scale of these changes was not as extreme as what is sometimes seen with *Wolbachia* transinfections in *Ae. aegypti* [10, 11, 14], the effect was still broad in scope and encompassed a wide range of biological processes, many of which are held in common with *Wolbachia* infections in other mosquitoes. Metabolic effects of *w*Flu infection appear to be particularly prominent [71], especially those associated with protein and lipid metabolism, and it is possible that the strain maintains the characteristic of a Jekyll and Hyde infection by both parasitising and providing key metabolites [26, 99]. And our results suggest that native strains such as *w*Flu likely have a greater impact on mosquito host biology than previously thought.

Critically, we did not see evidence that *w*Flu infection activated or suppressed the immune pathways typically associated with *Wolbachia* or *Plasmodium* infection [100, 101]. However, we did observe changes to a range of genes involved in immunity, oxidative stress and metabolism that have previously been associated with *Plasmodium* infection [59, 102], and could feasibly play a role in the enhancement of *P. gallinaceum*. Further molecular changes contributing to enhancement could be induced by infection or blood feeding, or under certain conditions associated with infections in the vertebrate host, and these could explain why enhancement of *P. gallinaceum* does not occur consistently [32]. *Plasmodium* enhancement is more prominent amongst transient artificial *Wolbachia* infections, where there is typically more extreme manipulation of host immunity and bacterial density that could contribute to the phenotype [103], and where temperature appears to be a key determinant [30]. While transient infections will likely never be utilised outside of the laboratory, determining the prevalence of enhancement amongst mosquito vectors naturally infected by *Wolbachia*, and identifying the causal mechanism of enhancement remain important issues going forward.

## Methods

### Mosquito material

Two mosquito lines were used in these experiments. The original, *Wolbachia*-infected *Aedes fluviatilis* (*w*Flu) line was derived from mosquitoes originally captured in Belo Horizonte in 1975 [32]. The mosquito colony was maintained in the laboratory until 2013 when a subset (Tet) was treated with tetracycline hydrochloride to remove the native *Wolbachia* infection, and then had their gut microbiota recolonized, as previously described [32]. Colony larvae were reared at low density in dechlorinated water, and were fed with fish food (Goldfish Colour, Alcon, Camboriú, Santa Catarina, Cat. No. 0504-2). Adults were maintained in low-density cages in a climate-controlled insectary (temperature: 27 ± 1 °C, RH: 70 ± 10%, photoperiod: 12 h light: 12 h dark), and provided 10% sucrose solution ad libitum. Mosquitoes used in experiments were maintained in small cylindrical cages (diameter – 16 cm, height – 18 cm) of approximately 80–90 individuals. Experiments were conducted more than 2 years after tetracycline treatment. In all experiments, the Tet line served as a *Wolbachia*-uninfected control line for the *w*Flu line, to study the effects of *Wolbachia* infection.

### RNA extractions & library preparation

Six groups of samples were prepared for sequencing, with each group going on to form an independent library. Three groups each of 16 6-day old whole adult females from the Flu and Tet lines were collected and total RNA extracted using the Trizol® protocol according to manufacturer’s instructions (Invitrogen), for a total of 6 independent samples, with 3 biological replicates per treatment. Mosquitoes were fed only 10% sucrose prior to collection. RNA levels in each sample were quantified using a NanoDrop ND1000 (ThermoFisher Scientific). Sample degradation levels were checked by running a portion of the samples on a standard non-denaturing agarose gel containing bleach [104].

cDNA libraries were constructed using Illumina Truseq RNA Sample Preparation Kit (Illumina Inc.) according to the manufacturer instructions starting with 4 μg of total RNA. The library products were then sequenced using an Illumina MiSeq platform on a paired-end 300 bp run. After cleaning reads from adaptor sequences, the quality of the reads was assessed using the FastQC program (http://www.bioinformatics.babraham.ac.uk/projects/fastqc/). Cleaned reads are available for download at the National Center for Biotechnology Information - Sequence Read Archive under the BioProject ID PRJNA320882.

### De novo transcriptome assembly and contig annotation

Since no reference genome was available for *Aedes fluviatilis*, a *de novo* transcriptome assembly was built with Trinity (https://github.com/trinityrnaseq/trinityrnaseq/wiki) using default parameters. All six Illumina RNAseq datasets were combined in order to assemble a more reliable transcriptome, with a total of 19,919,299 paired-end, high quality reads. Contig sequences were searched for candidate proteins with TransDecoder (https://transdecoder.github.io/), again using standard parameters. The assembled contigs were annotated through local alignments with BlastX (http://blast.ncbi.nlm.nih.gov/Blast.cgi) to the NCBI non-redundant (NR) and KEGG databases. BlastX parameters were set with an -e value of 1e-10. Blast2GO (https://www.blast2go.com/) was used to retrieve Gene Ontologies to annotated transcripts. Phylogenetic and divergence analyses were conducted using sequence data obtained during this study, or from UniProt (www.uniprot.org) or VectorBase (https://www.vectorbase.org). Methods and references for these analyses are described in Additional file 1.

### Read mapping and differential gene expression

All Illumina paired-end reads libraries were mapped separately against the *Ae. aegypti* predicted transcriptome, available at VectorBase, and the Trinity assembled contigs, both with Bowtie2 (http://bowtie-bio.sourceforge.net/bowtie2/index.shtml) using the default parameters while configuring fragment length. The Integrative Genomics Viewer (http://www.broadinstitute.org/igv/) was used to visualize the reads that were mapped back to the assembled transcriptome. Read counts mapped to each transcript were acquired with a custom Pearl script (available upon request).

Two R packages from Bioconductor (www.bioconductor.org), baySeq and DESeq2, were selected in order to identify the contigs that were significantly differentially expressed due to the presence of *Wolbachia*. The baySeq method is more sensitive, but also carries a greater false positive call rate, hence differential expression was confirmed using DESeq2 [45, 105]. The baySeq FDR (false discovery rate) *P* value for multiple tests was set to 0.05. The DESeq2 adjusted *P* value was set to 0.01, and transcripts were filtered according to their log2FoldChange (higher than +1 or lower than -1). We then obtained an estimate of the average fold change for each of the differentially expressed contigs using FPKM (fragments per kilobase of exon per million fragments mapped). Contigs that were differentially expressed but had no BlastX hit were excluded from analysis.

GO terms for the remaining contigs were generated through Blast2GO (https://www.blast2go.com) and VectorBase, or, when data could not be discovered from these sources, through FlyBase (http://flybase.org) or InterPro (http://www.ebi.ac.uk/interpro/), based on the analysis of homologous genes. GO information from contigs that matched to the same BlastX hit was considered only once, to avoid double counting. These GO lists were compiled and used to create two lists, one for the Flu libraries with the native *w*Flu *Wolbachia* infection, and the other for the Tet libraries, where the *Wolbachia* had been removed. Contigs were grouped according to putative function, and these lists were then compared to develop a profile of the transcriptomic effects of *w*Flu on its mosquito host.

Differentially expressed contig functions and GOs were then compared against the transcriptomic data from *w*Mel and *w*MelPop-infected *Aedes aegypti* arrays [11, 14], in order to develop a profile of types of transcriptional changes held in common between the three *Wolbachia* strains and their mosquito hosts. Contigs that had BlastX matches against *Ae. aegypti* were compared directly with *Ae. aegypti* expression data. Those contigs that matched to another species were compared directly against the *Ae. aegypti* genome in NCBI using BlastN (http://blast.ncbi.nlm.nih.gov/Blast.cgi). Any hits with a substantial match percentage (>80%) and a significant *e-value* were used for further analysis, using the first hit in a comparison in VectorBase while those that did not were not considered. This information was used to determine if any of the specific genes affected by *w*Flu infection were also affected by *w*Mel or *w*MelPop infection.

### Confirmation of differential expression

To assess the accuracy of the transcriptomic data set, six contigs were selected at random for expression analysis with RT-qPCR. Two of these contigs were indicated to have higher expression for Flu mosquitoes (AF10645; comp10645_c1_seq2 and AF10453; comp10453_c0_seq5), two had higher expression in Tet mosquitoes (AF15178; comp15178_c0_seq1 and AF14155; comp14155_c0_seq1), while two had equivalent expression between Flu and Tet mosquitoes (AF2025; comp2025_c0_seq1 and AF2041; comp2041_c0_seq1). The first four of these contigs were predicted to be differentially expressed through both the bayseq and DESeq2 analyses. Primers for these contigs were designed from the sequences generated during sequencing, which meant that they were suitable for cDNA. Primer sequences were designed using Primer 3 V0.4.0 (http://bioinfo.ut.ee/primer3-0.4.0/) to have a Tm of 55–60 °C and a product size range of 80–120 bp (Additional file 6).

Mosquitoes from both the Flu and Tet lines were reared as described above, and then females were collected individually at 6 days post eclosion. Total RNA was extracted and quantified as described above. First strand cDNA synthesis was conducted with 1 μg of total RNA from each sample using the M-MLV reverse transcriptase assay according to manufacturer’s instructions (Promega cat: C118A). cDNAs were then diluted 1:10 in nuclease-free water and stored at -30 °C. SYBR-based PCR was used to confirm the expression of each of the test genes, with 15 samples tested per mosquito line. Each gene was quantified in duplicate for all samples using the following mix: SYBR -5 μL, forward and reverse primers (10 μM) - 0.5 μL each, sterile RNase free water - 2 μL, sample 2 μL). RT-qPCR for samples was run on a LightCycler® 96 System (Roche) using the following profile: 10 min pre-incubation at 95 °C, 40 cycles of 15 s at 95 °C, 60 s at 60 °C, melt curve - 95 °C for 15 s, ramp from 60 °C to 95 °C at 1.6 °C/s. Expression values for each gene were normalised against *actin1* expression, which had previously been demonstrated to be a good control gene for *Ae. fluviatilis* [32]. Mean normalised expression values for each gene were calculated using Q-Gene [106], and were compared statistically between Flu and Tet mosquitoes using Mann Whiney U tests (Prism v 6.0 g, Graphpad).

## Additional files

Additional file 1: Divergence and phylogenetic analyses. Phylogenetic trees, and an estimate of the time of divergence for *Aedes fluviatilis* and other key mosquito species. (DOCX 1815 kb)
Additional file 2: Contigs of *Wolbachia* origin. List of contigs identified as being from *Wolbachia pipientis*. (XLSX 59 kb)
Additional file 3: Annotated, differentially expressed *Ae. fluviatilis* contigs. Table depicting all differentially expressed contigs, relevant annotations, and expression data. (XLSX 55 kb)
Additional file 4: GO terms with multiple hits associated with *w*Flu infection. Table depicting GO terms that were associated with more than one differentially expressed contig, for *Wolbachia*-infected and -uninfected mosquitoes. (DOCX 124 kb)
Additional file 5: Comparison of the transcriptomic effects of *w*Flu with *w*Mel and *w*MelPop infection in *Ae. aegypti.* Two tables. The first depicts gene homologs that are differentially expressed in *w*Flu-infected *Aedes fluviatilis* and/or *w*Mel-infected *Aedes aegypti*, and *w*MelPop-infected *Aedes aegypti*. The second depicts gene functions that are commonly differentially expressed between the three associations. (DOCX 154 kb)
Additional file 6: List of primers used for RT-qPCR confirmation of differential expression. Oligonucleotide sequences for genes used in this work. (DOCX 63 kb)

## Abbreviations

ATP: Adenine triphosphate
bp: Base pairs
CI: Cytoplasmic incompatibility
DAG: Diacylglyceride
FPKM: Fragments per kilobase of transcript per million mapped reads
GO: Gene ontology
Kb: Kilobase
KEGG: Kyoto encyclopedia of genes and genomes
KO: Kegg orthology
NR: NCBI non-redundant database
RNA-Seq: RNA sequencing (whole transcriptome shotgun sequencing)
ROS: Reactive oxygen species
rRNA: Ribosomal RNA
RT-qPCR: Quantitative reverse transcriptase PCR
snRNA: Small nuclear RNA
