# Supplementary material for: The transcriptome of the mosquito Aedes fluviatilis (Diptera: Culicidae), and transcriptional changes associated with its native Wolbachia infection
Source: BMC Genomics. 2017 Jan 3;18:6. doi: 10.1186/s12864-016-3441-4 (PMC5210266; doi:10.1186/s12864-016-3441-4)
Supplement: Additional file 1: — Divergence and phylogenetic analyses. Phylogenetic trees, and an estimate of the time of divergence for Aedes fluviatilis and other key mosquito species. (DOCX 1815 kb) [file 12864_2016_3441_MOESM1_ESM.docx]

**Additional File 1: Divergence and phylogenetic analyses**

This file describes the analyses used to characterize the phylogenetic relationship between *Aedes fluviatilis* and other key mosquito species, and those analyses used to estimate the time of divergence of *Aedes fluviatilis* from those species.

*
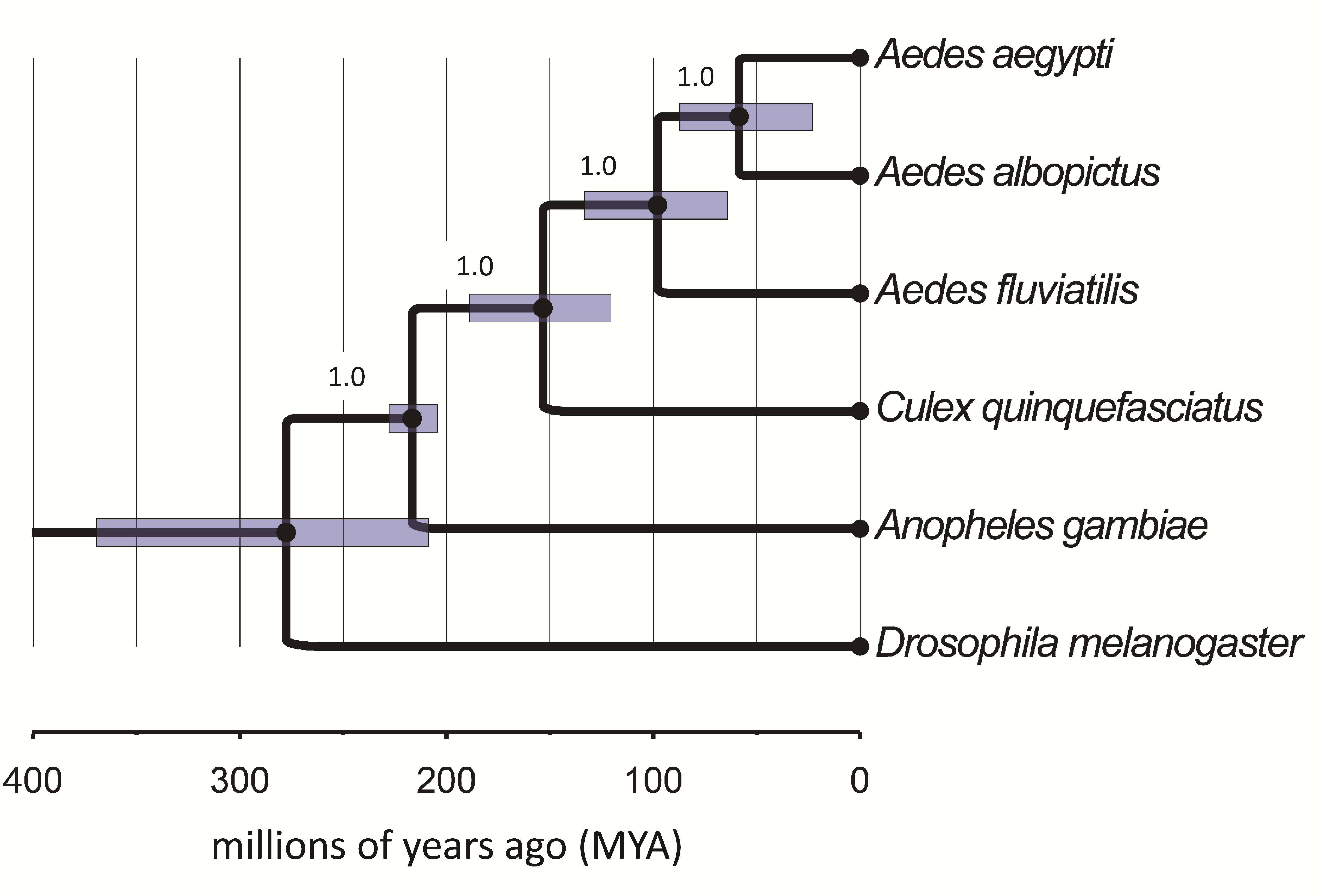
*

**Figure S1: Divergence time estimation and phylogeny for *Aedes fluviatilis* and other mosquito species.** The maximum clade credibility tree for *Aedes fluviatilis* and 4 other mosquito species estimated by Bayesian MCMC analysis using BEAST2. The numbers above the branches indicate, for their respective nodes, the posterior clade support. The node bars indicate the 95% highest posterior density (HPD) intervals for each of the node divergence time estimates. The analysis indicates that *Ae. fluviatilis* diverged from the other mosquito species approximately 98.0 MYA (95% HPD: 64.1 to 133.5 MYA).

**Figure S2: Phylogeny of mosquito species based on COX1 sequences.** Phylogenetic analysis through Bayesian inference for *Aedes fluviatilis*, 14 other mosquito species and *Drosophila melanogaster* based on the PF00115 conserved domain of the COX1 gene sequence.

*Divergence time estimation*

The divergence times and phylogeny of mosquitoes were estimated using a standard concatenated (“supermatrix”) analysis conducted in BEAST version 2.4.3 [1]. Nine single-copy and full-length (or nearly so) protein-coding loci – three mitochondrial (*coi*, *cytb* and *nd4*) and six nuclear (*ef1a*, *g6pd*, *gapdh*, *rpII215* and *rpL32*) – were used: in total 12,924 nucleotides, with mean 2585, and range 405 to 3,838, nucleotides per locus. The sequences for *Ae. fluviatilis* were taken from the assembled transcriptome reported here, while the orthologous sequences for other species were obtained from VectorBase (https://www.vectorbase.org) [2].

The sequences were manipulated and aligned using MAFFT version 7.222 [3] implemented in Geneious^©^ version 9.1.4 [4]. The XML command file containing the sequence data and model specification was created using BEAUti version 2.4.3 [5]. Unlinked site models were used for each locus, while the clock and tree models for all nine loci were linked. jModelTest version 2.1.7 [6] was used to determine and select according to AIC the best fitting substitution model for each locus. An uncorrelated lognormal relaxed clock was used [7], together with the calibrated Yule tree prior [8].

The clade of mosquitoes was constrained to be monophyletic, and the age of their common ancestor, based on a previously-derived and independent divergence time estimate [9], was specified using a normally-distributed calibration prior set with a mean of 216.86 MYA ± 6 standard deviations to give a 95% interval of approximately 205 to 228 MYA. The default values were used for all the other prior and operator settings. Four independent MCMC runs, each using the same sequence data and model settings, but different initial random number seeds, were performed. For each run, the MCMC was sampled every 2,000 generations for 6,000,000, with the first 500 (16.7%) samples discarded as burn-in. Convergence and mixing (i.e., stationarity) of the MCMC chains from each of the independent runs were checked and compared using Are We There Yet? (AWTY) [10] and Tracer version 1.6 [11].

The posterior samples (log and tree files) from each run minus burn-in (2,500 samples for each run) were combined using LogCombiner to give a final combined posterior sample size of 10,000. DensiTree version 2.1.11 [12] was used to explore the posterior tree sets, and TreeAnnotator version 2.4.2 was used to extract the maximum clade credibility tree and its associated posterior parameter estimates. The final dated tree was drawn and annotated using FigTree version 1.4.3 (Fig. S1A) [13].

*COX1 phylogenetic tree*

A second phylogenetic analysis was conducted with a dataset containing cytochrome oxidase subunit I (COX I) gene sequences retrieved from the Uniprot database (www.uniprot.org/). COX1 homologues from multiple mosquito species from the genera Anopheles, Aedes, Culex, and Ochlerotatus were retrieved by searching for the diagnostic domain (PF00115) according to the functional annotation at Pfam database [14]. Sequences were aligned with MAFFT version 7 (L-INS-i method).

The phylogenetic analysis was carried through Bayesian inference using the Markov Chain Monte Carlo method, as implemented in MrBayes version 3.2.5 [15], and according to [16]. Briefly, MrModeltest [17] was run with PAUP version 4.0a150 [18] to choose the model parameters to be estimated by Bayesian analyses using the Akiake Information Criterion (AIC) [19]. One cold and three heated chains were processed for 10-e7 generations, starting from a random tree with random parameters (topology, branch lengths and model parameters). These parameters were sampled every 1,000 generations, resulting in 10-e4 samples. After running the chains, we burned-out 25% of the first generations, checked for chain convergence (< 0.01) and used the remaining topologies (7,500) to build a majority-rule consensus tree. The consensus tree was drawn with FigTree [13], and edited using INKSCAPE (https://inkscape.org/en/).

**References**

1. Bouckaert R, Heled J, Kuhnert D, Vaughan T, Wu CH, Xie D, Suchard MA, Rambaut A, Drummond AJ: **BEAST 2: a software platform for Bayesian evolutionary analysis**. *PLoS Comput Biol* 2014, **10**(4):e1003537.

2. Giraldo-Calderon GI, Emrich SJ, MacCallum RM, Maslen G, Dialynas E, Topalis P, Ho N, Gesing S, VectorBase C, Madey G *et al*: **VectorBase: an updated bioinformatics resource for invertebrate vectors and other organisms related with human diseases**. *Nucleic Acids Res* 2015, **43**(Database issue):D707-713.

3. Katoh K, Standley DM: **MAFFT multiple sequence alignment software version 7: improvements in performance and usability**. *Mol Biol Evol* 2013, **30**(4):772-780.

4. Drummond AJ, Ashton B, Buxton S, Cheung M, Cooper A, Duran C, Heled J, Kearse M, Markowitz S, Moir R *et al*: **Geneious**. Version 9.1.4. Auckland, New Zealand: Biomatters Ltd; 2016.

5. Drummond AJ, Suchard MA, Xie D, Rambaut A: **Bayesian phylogenetics with BEAUti and the BEAST 1.7**. *Mol Biol Evol* 2012, **29**(8):1969-1973.

6. Darriba D, Taboada GL, Doallo R, Posada D: **jModelTest 2: more models, new heuristics and parallel computing**. *Nat Methods* 2012, **9**(8):772.

7. Drummond AJ, Ho SY, Phillips MJ, Rambaut A: **Relaxed phylogenetics and dating with confidence**. *PLoS Biol* 2006, **4**(5):e88.

8. Heled J, Drummond AJ: **Calibrated tree priors for relaxed phylogenetics and divergence time estimation**. *Syst Biol* 2012, **61**(1):138-149.

9. Reidenbach KR, Cook S, Bertone MA, Harbach RE, Wiegmann BM, Besansky NJ: **Phylogenetic analysis and temporal diversification of mosquitoes (Diptera: Culicidae) based on nuclear genes and morphology**. *BMC Evol Biol* 2009, **9**:298.

10. Nylander JA, Wilgenbusch JC, Warren DL, Swofford DL: **AWTY (are we there yet?): a system for graphical exploration of MCMC convergence in Bayesian phylogenetics**. *Bioinformatics* 2008, **24**(4):581-583.

11. Rambaut A, Suchard MA, Xie D, Drummond AJ: **Tracer**. In*.*, 1.6 edn; 2014.

12. Bouckaert RR: **DensiTree: making sense of sets of phylogenetic trees**. *Bioinformatics* 2010, **26**(10):1372-1373.

13. Rambaut A: **FigTree: Tree Figure Drawing Tool**. Version 1.4.3. University of Edinburgh, UK: Institute of Evolutionary Biology; 2016.

14. Finn RD, Coggill P, Eberhardt RY, Eddy SR, Mistry J, Mitchell AL, Potter SC, Punta M, Qureshi M, Sangrador-Vegas A *et al*: **The Pfam protein families database: towards a more sustainable future**. *Nucleic Acids Res* 2016, **44**(D1):D279-285.

15. Ronquist F, Teslenko M, van der Mark P, Ayres DL, Darling A, Hohna S, Larget B, Liu L, Suchard MA, Huelsenbeck JP: **MrBayes 3.2: efficient Bayesian phylogenetic inference and model choice across a large model space**. *Syst Biol* 2012, **61**(3):539-542.

16. Pylro VS, Morais DK, Kalks KH, Roesch LF, Hirsch PR, Totola MR, Yotoko K: **Misguided phylogenetic comparisons using DGGE excised bands may contaminate public sequence databases**. *J Microbiol Methods* 2016, **126**:18-23.

17. Nylander JA: **MrModeltest**. Version 2. Uppsala University, Sweden: Evolutionary Biology Centre; 2004.

18. Swofford DL: **PAUP*. Phylogenetic Analysis Using Parsimony (*and other methods)**. Version 4. Sunderland, Massachusetts: Sinauer Associates; 2003.

19. Posada D, Buckley TR: **Model selection and model averaging in phylogenetics: advantages of akaike information criterion and bayesian approaches over likelihood ratio tests**. *Syst Biol* 2004, **53**(5):793-808.
