# Supplementary material for: The transcriptome of the mosquito Aedes fluviatilis (Diptera: Culicidae), and transcriptional changes associated with its native Wolbachia infection
Source: BMC Genomics. 2017 Jan 3;18:6. doi: 10.1186/s12864-016-3441-4 (PMC5210266; doi:10.1186/s12864-016-3441-4)
Supplement: Additional file 4: — GO terms with multiple hits associated with wFlu infection. Table depicting GO terms that were associated with more than one differentially expressed contig, for Wolbachia-infected and -uninfected mosquitoes. (DOCX 124 kb) [file 12864_2016_3441_MOESM4_ESM.docx]

**Additional File 4: GO terms with multiple hits associated with *w*Flu infection**

| **Upregulated contigs** | | **Downregulated contigs** | |
| --- | --- | --- | --- |
| **GO** | **Hits** | **GO** | **Hits** |
| GO:0016021, integral to membrane | 12 | GO:0016021, integral to membrane | 10 |
| GO:0055085, transmembrane transport | 8 | GO:0007186, G-protein coupled receptor protein signalling pathway | 8 |
| GO:0055114, oxidation-reduction process | 7 | GO:0004930, G-protein coupled receptor activity | 7 |
| GO:0003676, nucleic acid binding | 6 | GO:0007601, visual perception | 6 |
| GO:0020037, heme binding | 6 | GO:0007602, phototransduction | 6 |
| GO:0015074, DNA integration | 5 | GO:0009881, photoreceptor activity | 6 |
| GO:0005634, nucleus | 5 | GO:0018298, protein-chromophore linkage | 6 |
| GO:0008270, zinc ion binding | 4 | GO:0003676, nucleic acid binding | 4 |
| GO:0006508, proteolysis | 4 | GO:0005515, protein binding | 4 |
| GO:0005975, carbohydrate metabolic process | 4 | GO:0005524, ATP binding | 3 |
| GO:0005524, ATP binding | 4 | GO:0005576, extracellular region | 3 |
| GO:0005488, binding | 4 | GO:0006508, proteolysis | 3 |
| GO:0004252, serine-type endopeptidase activity | 4 | GO:0015074, DNA integration | 3 |
| GO:0046872, metal ion binding | 3 | GO:0000166, nucleotide binding | 2 |
| GO:0016705, oxidoreductase activity, acting on paired donors, with incorporation or reduction of molecular oxygen | 3 | GO:0003677, DNA binding | 2 |
| GO:0016020, membrane | 3 | GO:0003924, GTPase activity | 2 |
| GO:0009055, electron carrier activity | 3 | GO:0004803, transposase activity | 2 |
| GO:0005576, extracellular region | 3 | GO:0005525, GTP binding | 2 |
| GO:0005506, iron ion binding | 3 | GO:0016787, hydrolase activity | 2 |
| GO:0004497, monooxygenase activity | 3 |  |  |
| GO:0048813, dendrite morphogenesis | 2 |  |  |
| GO:0044446, intracellular organelle part | 2 |  |  |
| GO:0044238, primary metabolic process | 2 |  |  |
| GO:0030983, mismatched DNA binding | 2 |  |  |
| GO:0030529, ribonucleoprotein complex | 2 |  |  |
| GO:0022857, transmembrane transporter activity | 2 |  |  |
| GO:0016787, hydrolase activity | 2 |  |  |
| GO:0016758, transferase activity, transferring hexosyl groups | 2 |  |  |
| GO:0016746, transferase activity, transferring acyl groups | 2 |  |  |
| GO:0008152, metabolic process | 2 |  |  |
| GO:0008061, chitin binding | 2 |  |  |
| GO:0007186, G-protein coupled receptor protein signaling pathway | 2 |  |  |
| GO:0006811, ion transport | 2 |  |  |
| GO:0006298, mismatch repair | 2 |  |  |
| GO:0005811, lipid particle | 2 |  |  |
| GO:0005525, GTP binding | 2 |  |  |
| GO:0005515, protein binding | 2 |  |  |
| GO:0004553, hydrolase activity, hydrolyzing O-glycosyl compounds | 2 |  |  |
| GO:0003924, GTPase activity | 2 |  |  |
