# Supplementary material for: The transcriptome of the mosquito Aedes fluviatilis (Diptera: Culicidae), and transcriptional changes associated with its native Wolbachia infection
Source: BMC Genomics. 2017 Jan 3;18:6. doi: 10.1186/s12864-016-3441-4 (PMC5210266; doi:10.1186/s12864-016-3441-4)
Supplement: Additional file 5: — Comparison of the transcriptomic effects of wFlu with wMel and wMelPop infection in Ae. aegypti. Two tables. The first depicts gene homologs that are differentially expressed in wFlu-infected Aedes fluviatilis and/or wMel-infected Aedes aegypti, and wMelPop-infected Aedes aegypti. The second depicts gene functions that are commonly differentially expressed between the three associations. (DOCX 154 kb) [file 12864_2016_3441_MOESM5_ESM.docx]

**Additional File 5: Comparison of the transcriptomic effects of *w*Flu with *w*Mel and *w*MelPop infection in *Ae. aegypti*.**

**A: Differentially expressed genes that are also affected by *w*Mel/*w*MelPop infection in *Ae. aegypti***

| **Upregulated with *w*Flu infection** |  | ***Ae. aegypti* match** | **AFC in *Ae. aegypti*** |
| --- | --- | --- | --- |
|  | | | |
| **Upregulated only with *w*Mel** | | | |
| comp4837_c0_seq1 | salivary basic peptide -1 | AAEL013170-RA | 1.1 |
| comp5926_c0_seq2 | monocarboxylate transporter | AAEL004213-RA | 1.4 |
| **Downregulated only with *w*Mel** | |  |  |
| comp11035_c0_seq1 | voltage-dependent para-like sodium channel | AAEL000009-RA | 0.77 |
| **Upregulated only with wMelPop** | | | |
| comp12058_c1_seq1 | o-linked n-acetyl glucosamine transferase | AAEL014617-RA | 1.8 |
| comp309_c0_seq1 | rho guanyl-nucleotide exchange factor | AAEL009966-RA | 1.8 |
| comp8292_c0_seq2 | galactose-specific c-type | AAEL007766-RA | 1.1 |
| comp10441_c0_seq1 | hypothetical protein AaeL_AAEL009476 | AAEL007815-RA | 1.6 |
| comp12611_c0_seq1 | beta-hexosaminidase b | AAEL004931-RB | 2.3 |
| comp14074_c0_seq1 | agap000603-pa-like protein | AAEL002908-RA | 1.6 |
| comp5400_c0_seq1 | synaptic vesicle protein | AAEL005375-RA | 1.8 |
| comp8899_c0_seq1 | hypothetical protein AaeL_AAEL000500 | AAEL000500-RA | 2.1 |
| comp9458_c0_seq1 | a chain crystal structure of beta-glucosidase | AAEL010935-RA | 1.1 |
| comp10525_c0_seq3 | brain chitinase and chia | AAEL003066-RA | 1.7 - 2.2 |
| comp2547_c0_seq1 | hypothetical protein AaeL_AAEL012837 | AAEL009476-RA | 1.9 - 4.8 |
| comp6505_c0_seq1 | glucose transport protein | AAEL002292-RA | 1.9 - 2.2 |
| comp7631_c0_seq3 | agap013493-pa-like protein | AAEL003190-RA | 1.8 - 3.0 |
| **Downregulated only with *w*MelPop** | | | |
| comp10103_c0_seq2 | neuferricin homolog, cytochrome b5 domain-containing protein 2 | AAEL009625-RA | 0.3 |
| comp11118_c0_seq1 | gram negative bacteria binding protein 2 | AAEL006598-RA | 0.56 |
| comp9289_c0_seq1 | serine threonine-protein kinase rio1 | AAEL005533-RA | 0.56 |
| comp10042_c0_seq1 | stretchin- isoform v | AAEL012837-RA | 0.53 |
| comp8312_c0_seq6 | gtp-binding protein di-ras2-like | AAEL013856-RA | 0.29 |
| **Upregulated with both *w*Mel and *w*MelPop** | | | |
| comp5863_c0_seq3 | AAEL017492-PA | AAEL013039-RA | 1.8 - 2.5 |
|  |  |  |  |
| **Downregulated with *w*Flu infection** |  | ***Ae. aegypti* match** | **AFC in *Ae. aegypti*** |
|  |  |  |  |
| **Downregulated only with *w*MelPop** | |  |  |
| comp4000_c0_seq1 | agap012985-pa-like protein | AAEL006498-RA | 0.77 |
| **Upregulated only with wMelPop** | |  |  |
| comp9621_c0_seq1 | blue-sensitive visual pigment | AAEL003035-RA | 1.4 |
| comp10513_c0_seq1 | juvenile hormone-inducible protein | AAEL004023-RA | 1.7 |
| comp10957_c0_seq1 | serine protease | AAEL006586-RA | 3.3 |
| comp12565_c0_seq3 | t-complex protein 1 subunit zeta | AAEL006946-RA | 1.2 |
| comp2653_c0_seq1 | mucin-like peritrophin | AAEL009219-RA | 1.7 |
| comp5123_c0_seq1 | serine protease 14 | AAEL000028-RB | 2.4 |
| comp5269_c0_seq1 | hypothetical protein AaeL_AAEL005768 | AAEL005768-RA | 1.4 |
| comp6296_c0_seq4 | short form d7 salivary protein sd7-1 | AAEL006423-RA | 1.4 |
| comp6651_c0_seq1 | short form d7 salivary protein sd7-1 | AAEL006423-RA | 1.4 |
| comp7785_c0_seq8 | isoform a | AAEL004042-RC | 1.1 |
| comp8245_c0_seq1 | isoform a | AAEL008485-RA | 1.5 |
| comp9869_c0_seq2 | agap002274-pb-like protein | AAEL011574-RA | 1.7 |
| comp10289_c0_seq6 | guanine nucleotide-binding protein subunit beta-2 | AAEL008108-RA | 1.4 |
| **Downregulated with both *w*Mel and *w*MelPop** | |  |  |
| comp8001_c0_seq1 | ultraviolet-sensitive opsin | AAEL009615-RA | 0.77 |
| comp14026_c0_seq1 | agap003775-pa-like protein | AAEL010180-RA | 0.25 - 0.42 |
| comp14516_c0_seq1 | zinc metalloprotease | AAEL008527-RA | 0.83 |
| comp3352_c0_seq1 | hypothetical protein AaeL_AAEL006662 | AAEL006662-RA | 0.14 - 0.63 |
| **Downregulated with *w*Mel but upregulated with *w*MelPop** | |  |  |
| comp7683_c0_seq1 | membrane glycoprotein lig-1 | AAEL008753-RA | 0.67/3.0 |
| comp7548_c0_seq2 | calmodulin-like protein 4-like | AAEL011979-RA | 0.77/1.2 - 1.4 |

**B: Gene functions affected by *w*Flu, *w*MelPop and *w*Mel in their respective mosquito hosts.**

| **Upregulated with *w*Flu infection** | | | |
| --- | --- | --- | --- |
|  | **Number of differentially expressed transcripts** | | |
| **Gene type** | ***w*MelPop** | ***w*Mel** | ***w*Flu** |
| Serine protease | 54 | 11 | 2 |
| Cytochrome p450 | 45 | 7 | 3 |
| Galactose-specific C-type lectin | 12 | 5 | 1 |
| Membrane transport protein | 12 | 1 | 1 |
| Glucosyl/glucuronosyl transferases | 10 | 0 | 2 |
| Chymotrypsin | 7 | 1 | 1 |
| Trypsin | 7 | 1 | 2 |
| Zinc finger protein | 7 | 3 | 2 |
| Odorant-binding protein | 5 | 1 | 1 |
| Brain chitinase and chia | 4 | 0 | 1 |
| Niemann-pick type c2 | 4 | 0 | 1 |
| Serine threonine-protein kinase | 4 | 2 | 1 |
| Short-chain dehydrogenase | 4 | 1 | 1 |
| Synaptic vesicle protein | 4 | 0 | 1 |
| CD36 antigen | 3 | 0 | 1 |
| Glucose transport protein | 3 | 0 | 1 |
| Gram negative bacteria binding protein 2 | 3 | 2 | 1 |
| Major allergen bla g | 3 | 0 | 1 |
| Ribosomal protein s10 | 3 | 0 | 1 |
| β-hexosaminidase B | 3 | 0 | 1 |
| Anterior fat body protein | 2 | 0 | 1 |
| Chorion peroxidase | 2 | 0 | 1 |
| Fasciculation and elongation protein ζ-2 | 2 | 0 | 1 |
| Monocarboxylate transporter | 2 | 0 | 1 |
| Neuferricin homolog, cytochrome b5 domain-containing protein 2 | 2 | 0 | 1 |
| Pancreatic triacylglycerol lipase | 2 | 1 | 1 |
| Permease | 2 | 1 | 1 |
| γ glutamyl transpeptidases | 2 | 0 | 1 |
| Acyltransferase | 1 | 0 | 2 |
| CDC42 protein | 1 | 0 | 1 |
| RNase H and integrase-like protein | 1 | 0 | 1 |
| Stretchin- isoform v | 1 | 0 | 1 |
| α chain crystal structure of β-glucosidase | 1 | 0 | 1 |

| **Downregulated with *w*Flu infection** | | | |
| --- | --- | --- | --- |
|  | **Number of differentially expressed transcripts** | | |
| **Gene type** | ***w*MelPop** | ***w*Mel** | ***w*Flu** |
| Cuticular protein isoform a | 9 | 1 | 1 |
| Vitellogenic carboxypeptidase | 9 | 1 | 1 |
| Zinc metalloprotease | 8 | 0 | 2 |
| Anterior fat body protein | 4 | 0 | 1 |
| Histone h1 | 2 | 0 | 1 |
| Serine protease | 2 | 0 | 2 |
| ABC transporter ATP-binding protein | 1 | 0 | 1 |
| Acyltransferase | 1 | 0 | 1 |
| Calmodulin-binding protein trpl | 1 | 0 | 2 |
| Galactose-specific C-type | 1 | 0 | 1 |
| Insulin receptor tyrosine kinase substrate | 1 | 0 | 1 |
| Membrane glycoprotein lig-1 | 1 | 0 | 1 |
| RNase h and integrase-like protein | 1 | 0 | 1 |

*Ae. aegypti* gene IDs from VectorBase (http://vectorbase.org). AFC - average fold change due to *w*Mel or *w*MelPop infection in *Ae. aegypti*. *Ae. aegypti* were collected through microarray analysis in previous studies:

Kambris Z, Cook PE, Phuc HK, Sinkins SP. Immune activation by life-shortening *Wolbachia* and reduced filarial competence in mosquitoes. Science. 2009;326(5949):134-6. doi: Doi 10.1126/Science.1177531. PubMed PMID: ISI:000270355600053.

Rancès E, Ye YH, Woolfit M, McGraw EA, O'Neill SL. The relative importance of innate immune priming in *Wolbachia*-mediated Dengue interference. Plos Pathogens. 2012;8(2). doi: e1002548.
