# Supplementary material for: The transcriptome of the mosquito Aedes fluviatilis (Diptera: Culicidae), and transcriptional changes associated with its native Wolbachia infection
Source: BMC Genomics. 2017 Jan 3;18:6. doi: 10.1186/s12864-016-3441-4 (PMC5210266; doi:10.1186/s12864-016-3441-4)
Supplement: Additional file 6: — List of primers used for RT-qPCR confirmation of differential expression. Oligonucleotide sequences for genes used in this work. (DOCX 63 kb) [file 12864_2016_3441_MOESM6_ESM.docx]

**Additional File 6: List of Primers used for RT-qPCR confirmation of differential expression**

All primers are listed 5’ - 3’.

**Actin-2_F:** GTCCGCGATATCAAGGAAAA

**Actin-2_R:** GTGTTGGCGTACAGGTCCTT

**AF2025_F:** GGTCTCACAGTAGTTGGCCT

**AF2025_R:** TCCTTGCGTATATTTGCGGC

**AF2041_F:** TCAAACACACATGAGCCAGC

**AF2041_R:** GTGAATGGGACTCGATTGCC

**AF10453_F:** GTCTACAATGGGCTGCAAGG

**AF10453_R:** ACTTCATGCACTCGAGTTGC

**AF10645_F:** CACCTTTCCCTCACGGTACT

**AF10645_R**: GGGCACGTGAAATCCTGTTT

**AF14155_F:** ATTTGCTGGGCTGGTTTACG

**AF14155_R:** ACTTGTTCGGACCCTTCATCA

**AF15178_F:** TGCTAGGCTCAGTTTCCCTT

**AF15178_R:** AACGCGTTTATGTCCTGGTG
